# Liver function parameters aspartate aminotransferase and total protein predict functional outcome in stroke patients with non-cardioembolism

**DOI:** 10.3389/fnut.2022.918553

**Published:** 2022-08-18

**Authors:** Jiali Xie, Yinmeng Zhu, Chunyang Pang, Lingfei Gao, Huan Yu, Wenjing Lv, Wanli Zhang, Binbin Deng

**Affiliations:** ^1^Department of Neurology, First Affiliated Hospital of Wenzhou Medical University, Wenzhou, China; ^2^First Clinical College of Wenzhou Medical University, Wenzhou, China; ^3^Department of Pediatrics, Second Affiliated Hospital and Yuying Children’s Hospital of Wenzhou Medical University, Wenzhou, China; ^4^Department of Geriatrics, The Affiliated Hospital of Qingdao University, Qingdao, China

**Keywords:** functional outcome, nomogram, non-cardioembolism, stroke highlight, liver function parameters

## Abstract

Stroke, classified as cardioembolism and non-cardioembolism (non-CE), entails a large socioeconomic burden on the elderly. The morbidity and mortality of non-CE are high, whereas studies concerning prognostic factors impacting function outcome remain underdeveloped and understudied. Liver function parameters are convenient approaches to predicting prognosis in cardiovascular diseases, but their clinical significance has not been well characterized in stroke, especially in non-CE. In our study, a total of 576 patients with non-CE at 1 year of follow-up were enrolled in a cohort from a consecutive hospital-based stroke registry, with randomly 387 patients as the development cohort and 189 patients as the validation cohort. The univariate and multivariate analyses revealed the following novel findings: (i) The incidence of unfavorable functional outcomes after non-CE was significantly greater (*p* < 0.01) in patients with higher age, aspartate aminotransferase (AST), the National Institutes of Health Stroke Scale (NIHSS) score, and depressed total protein (TP); (ii) We established a novel model and nomogram to predict stroke prognosis, in addition to the known factors (age and the NIHSS score). The levels of AST and TP were independently correlated with the incidence of unfavorable outcomes [AST: odds ratio (OR) = 1.026, 95% CI (1.002–1.050); TP: OR = 0.944, 95% CI (0.899–0.991)]; (iii) The results persisted in further subgroup analysis stratified by age, gender, the NIHSS score, and other prespecified factors, especially in males 60 years or older. Overall, this study demonstrates that hepatic parameters (AST and TP) after non-CE are considered to be associated with functional outcomes at 1-year follow-up, especially in males aged ≥ 60 years.

## Highlights

- First, this is the first study to disentangle the correlation between liver function indicators and functional outcomes in patients diagnosed with non-cardioembolism (non-CE).
- Second, increased aspartate aminotransferase (AST) levels and decreased total protein (TP) levels were investigated in patients with non-CE with unfavorable functional outcomes.
- Third, despite the already known predictive biomarkers (age and the NIHSS score), serum concentrations of AST and TP are correlated with stroke prognosis, especially in males aged ≥ 60 years.

## Introduction

Stroke, still the second leading cause of death (1), yields high morbidity and mortality worldwide (2). Given the accelerated aging process, the incidence of stroke is increasing gradually, conferring a rigorous threat to the health of the elderly (3). Currently, the gold standard for stroke treatment is reperfusion therapy, including intravenous recombinant tissue plasminogen activator (rt-PA) and intravascular therapy (4). The mortality of stroke has remained high over the past four decades despite existing treatments (5).

A multitude of empirical studies has investigated prognostic factors impacting stroke, incorporating age, stroke etiology, history of hypertension, and diabetes mellitus (1, 3, 6). Of note, stroke severity keeps the most powerful biomarker for the functional prognosis (7). Identifying prognostic indicators in acute ischemic stroke (AIS) enables physicians to build evidence-based treatments and confer more valid interventions involved in AIS.

To date, a bulk of research has unraveled that hepatic parameters serve as well-documented indicators for cardiovascular diseases (8–12), yet the association between hepatic parameters and stroke prognosis has performed mixed findings. There is growing evidence that insufficient albumin concentration was involved in the death of all-cause and cardiovascular diseases (12, 13). It has been further investigated that serum albumin was inversely related to the risk of both the cardiogenic and cryptogenic stroke (14). Several subsequent studies have convincingly confirmed that in non-alcoholic liver diseases and other liver diseases, the levels of alanine aminotransferase (ALT), aspartate aminotransferase (AST), and gamma-glutamyl transpeptidase (GGT) were ascending and considered to exert correlations with cardiovascular events (8, 15), as well as stroke (16). Research based on the Japanese population highlighted whether GGT was correlated with the risk of stroke and concluded similar results, but only in women (17). Wang et al. demonstrated that in 73 patients with large artery atherosclerosis stroke, both the direct and indirect bilirubin levels were positively linked to stroke severity on the 1st, 7th, and 14th day and unfavorable functional outcomes on the 30th day (18).

According to the Trial of ORG 10,172 in Acute Stroke Treatment (TOAST) (19), AIS can be subdivided into five classes: large artery atherosclerosis, cardioembolism, small-artery occlusion, other determined etiology, and undetermined etiology. Stroke can also be divided into cardioembolism and non-cardioembolism (non-CE). As a special cause of stroke, cardioembolism has been extensively explored. There exist higher morbidity and mortality in non-CE; however, relevant studies regarding risk factors impacting functional outcomes in non-CE are still obscure. In a bid to minimize the interference of cardiogenic factors, herein, we enrolled patients diagnosed as non-CE to minimize the complications’ influences of cardiovascular events on liver function indicators.

Thus, this prospective study, focusing on 567 patients with non-CE, further disentangled the link between liver function indicators and functional outcomes after stroke. We, herein, postulated that hepatic parameters constituted crucial elements for stroke prognosis in patients hospitalized for non-CE. There exist a host of studies establishing nomograms with regard to the prognosis of diseases, whereas the nomograms in stroke outcomes remain largely insufficient. Hence, a nomogram built on hepatic parameters was established here.

## Materials and methods

### Subjects and setting

The study protocol was approved by the Ethics Committee of the First Affiliated Hospital of Wenzhou Medical University and complied with the Declaration of Helsinki. The period of recruitment of the patients was carried out from December 2019 to December 2020, and 1,019 patients with AIS with non-CE were obtained. The exclusion criteria for patients diagnosed with non-CE constituted: (1) patients with a history of cerebral hemorrhage, subarachnoid hemorrhage, or cerebral venous thrombosis; (2) lack of outcome variables after 1-year follow-up; and (3) any factors that affect liver function indicators, comprising active infection, immunosuppressant or antibiotic use before admission, as well as recent trauma or major surgery.

A total of 567 patients with non-CE were included through the inclusion and exclusion criteria and all the subjects signed informed consent. The approach for selecting two-thirds to fourth-fifths of individuals as the development group is quite common (20–22). Using the R package of the R version, randomly, we allocated two-thirds of patients (*n* = 378) as the development group and one-third of patients (*n* = 189) as the validation group.

Medical history recording of blood samples was performed on the morning of the second day of admission after overnight fasting. Carotid ultrasonography and CT/MRI were carried out after admission. Before discharge, CT and MRI were reassessed to make a final diagnosis. The stroke subtype was determined according to the TOAST criteria (19). Additionally, the National Institutes of Health Stroke Scale (NIHSS) scores were assessed on admission to evaluate the stroke severity.

Laboratory data collected included: (1) demographic characteristics (age and gender); (2) clinical characteristics (the NIHSS on admission, blood pressure, history of smoking, and drinking alcohol); (3) past medical history (hypertension, diabetes mellitus, and atrial fibrillation); (4) hepatic function parameters [total protein (TP), albumin (ALB), ALT, AST, alkaline phosphatase (ALP), GGT, total bilirubin (TB), direct bilirubin (DB), and indirect bilirubin (IB)]; and (5) blood biochemistry, including lymphocyte, neutrophil, neutrophil-to-lymphocyte ratio (NLR), platelet (PLT), red blood cell (RBC), hemoglobin (HB), creatinine (Cr), total cholesterol (TC), high-density lipoprotein cholesterol (HDL-C), low-density lipoprotein cholesterol (LDL-C), blood urea nitrogen (BUN), urea creatinine ratio (BUN/Cr), thyroid-stimulating hormone (TSH), homocysteine (Hcy), thyroid hormones (THs), free triiodothyronine (fT3), free tetraiodothyronine (fT4), fibrinogen (FI), and creatine kinase (CK).

### Follow-up

Clinical outcomes were further assessed after 3 months and 1 year. Each participant underwent a minimum of 1-year follow-up *via* telephone, email, questionnaires, and outpatient reviews. Independent investigators were blinded to the clinical data and neuroimaging evaluations.

### Patient assessment

The Modified Rankin Scale (mRS) was utilized to evaluate stroke prognosis after 3 months and 1 year of follow-up (23). The mRS score of 0–2 was categorized as a favorable outcome, whereas the mRS score of 3–6 was manifested as an unfavorable outcome.

### Statistical analysis

Categorical variables were expressed as frequency and percentage (N,%) using the chi-squared test or Fisher’s exact test, and continuous variables were expressed as mean ± SD or median and quartile ranges [median, interquartile range (IQR)] using the *t*-test or Mann–Whitney *U* test. The Spearman’s or Pearson’s analysis was utilized to test the correlation between variables. Screened in the univariate analysis, factors with *p* < 0.05 were then incorporated into the multivariate logistic regression analysis to identify independent predictors. Additionally, three models were constructed and the receiver operating characteristic (ROC) curves, the integrated discrimination improvement (IDI), the net reclassification index (NRI), and the decision curve analysis (DCA) values were calculated to compare the predictive power of different models. The nomogram and calibration curves, performed with package rms in the R version, were established based on such analysis. Finally, subgroup analysis was performed to further verify the reliability and practicability of our results, and the interaction was tested. A value of *p* < 0.05 was deemed to be statistically crucial. All the calculations were carried out using SPSS version 25.0 software and R version 4.1.0.

## Results

### Baseline characteristics of study participants

During the study period, a total of 567 patients with non-CE were screened, with 378 patients as the development group and 189 patients as the validation group. The baseline characteristics of the total and development cohorts are shown in Supplementary Table 1. Of all the patients with non-CE in the development cohort, 292 (77.2%) participants experienced good functional outcomes (mRS ≤ 2) and 86 (22.8%) participants developed poor functional outcomes (mRS > 2). In patients attributable to good functional outcomes, the mean age was 64.54 years ranging from 53 to 76 years, with 60.6% male predominance. Furthermore, in the group with poor functional outcome, the mean age was 69.33 years ranging from 59 to 80 years, with 69.8% male predominance. We confirmed that, compared with patients with good functional outcomes, patients with poor functional outcomes had the higher NIHSS score, NLR, AST, FI, and elder age, whereas they had lower levels of TP, ALB, and fT3 (*p* < 0.05).

### Difference of liver function indicators in stroke prognosis (modified rankin scale)

**FIGURE 1:**
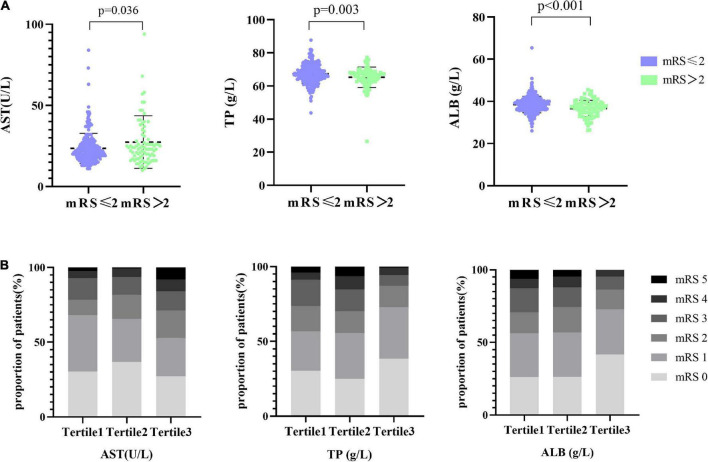
Distribution of AST and TP with the different mRS groups. **(A)** The scatter diagram in the distribution of AST, TP, and ALB between the mRS ≤ 2 and mRS > 2 groups, respectively. **(B)** Functional outcome at 1 year based on the mRS, presented as percentage and stratified by AST, TP, and ALB levels. Patients were divided into the three groups according to the tertiles of AST (AST ≤ 19 U/l, 19 U/l < AST ≤ 25 U/l, AST > 25 U/l), the tertiles of TP (TP ≤ 64.9 g/l, 64.9 g/l < TP ≤ 68.7 g/l, TP > 68.7 g/l), and the tertiles of ALB (ALB ≤ 36.7 g/l, 36.7 g/l < ALB ≤ 39.4 g/l, ALB > 39.4 g/l).

In the univariate analysis, we obtained that liver function indicators, including AST, TP, and ALB, were statistically different (*p* = 0.036, *p* = 0.003, and *p* < 0.001, respectively) in stroke prognosis (Figure 1A). Furthermore, the distributions of the mRS scores stratified by the tertiles of AST, TP, and ALB levels are given in Figure 1B. With an increase of AST levels, the proportion of the mRS ≥ 2 was prone to be elevated (from 32, 34.4 to 47.2%). Nevertheless, the proportion of the mRS 2–3 descended in elevated TP levels and the proportion of the mRS 3–5 decreased in higher ALB levels. We performed that serum levels of AST (*r* = 0.137, *p* = 0.008), TP (*r* = −0.147, *p* = 0.004), and ALB (*r* = −0.225, *p* < 0.001) at admission had statistically significant correlations with the mRS after 1-year follow-up (Supplementary Figures 1A–C). The heat map was utilized to alleviate the collinearity of the model in the Spearman’s correlation analysis (Supplementary Figure 1D). We explored that ALB was weakly associated with age and moderately correlated with TP, and, consequently, all of them were relatively independent (*r* < 0.8) and were included in our model.

### Correlation of liver function parameters with functional outcome

**FIGURE 2:**
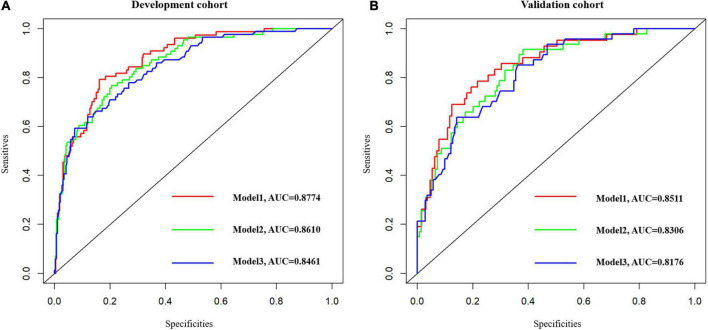
The ROC curves of the three models. Model 1: NIHSS + Age + AST + TP + ALB + NLR + fT3 + FI. Model 2: NIHSS + Age + Hepatic function parameters (AST, TP). Model 3: NIHSS + Age. **(A)** Model 1 vs. model 2, *Z* = 0.1756, *p*-value = 0.8607. **(B)** Model 1 vs. model 2, *Z* = 1.4837, *p*-value = 0.1379. ROC, receiver operating characteristic.

Baseline factors with *p* < 0.05, constitutive of age, the NIHSS, AST, TP, ALB, NLR, fT3, and FI in Supplementary Table 1 were obtained in model 1. Ultimately, we considered the age of patients [odds ratio (OR): 1.034, 95% CI: 1.022–1.067], the NIHSS on admission (OR: 1.613, 95% CI: 1.423–1.828), TP (OR: 0.918, 95% CI: 0.854–0.986), and AST (OR: 1.029, 95% CI: 1.003–1.056), a total of four variables as independent factors on the unfavorable functional outcome as evaluated by the mRS > 2 at 1 year (Supplementary Table 2). Consistent with our results, we revealed that age (OR: 1.067, 95% CI: 1.019–1.118), the NIHSS on admission (OR: 1.734, 95% CI: 1.421–2.115), TP (OR: 0.898, 95% CI: 0.809–0.998), and AST (OR: 1.042, 95% CI: 1.002–1.085) were served as crucial predictors on functional outcome at 3-month follow-up (Supplementary Table 3). In a bid to verify whether liver function indicators (AST and TP) would improve the capacity to predict stroke prognosis, two additional prediction models based on liver function indicators have been constructed: model 2: NIHSS + age + hepatic parameters (AST and TP) and model 3: NIHSS + age; the ROC curves for functional outcome are shown in Figure 2 and the three area under the curves (AUCs) to discriminate functional outcome in the development group were 0.8774, 0.8610, and 0.8461, respectively. Also, the AUC of the adjusted model for functional outcomes at 3-month follow-up was 0.8753, which showed good discrimination (Supplementary Figure 2). In the validation group, we discovered that the AUCs of the three ROCs reached 0.8511, 0.8306, and 0.8176, respectively. What is more, the difference of the AUCs was indiscriminate between model 1 and model 2 in the development group (*Z* = 0.1756, *p*-value = 0.8607) as well as in the validation group (*Z* = 1.4837, *p*-value = 0.1379).

### Predictive values of liver function parameters for functional outcome

Decision curve analysis, performed to test the clinical discriminative ability of three models, observed that the net benefits afforded by model 1 and model 2 were superior to model 3 (Supplementary Figure 3). NRI and IDI are calculated concerning model 2 and model 3, as shown in Supplementary Table 4 and Supplementary Figure 4. We observed that model 2, the addition of AST and TP based on model 3, did not statistically ascend the discriminatory power, but significantly increases the risk reclassification for functional outcome in patients with non-CE, as observed by the category-free NRI of 0.1141% (95% CI 0.0260–0.2021%; *p* = 0.0111) and IDI of 0.0228% (95% CI −0.0011 to −0.0467%; *p* = 0.0611) in the development group. Additionally, similar results were obtained in the validation cohort (NRI of 0.1423%, 95% CI 0.0122–0.2724%; *p* = 0.0321 and IDI of 0.0206%, 95% CI −0.0008 to 0.0421%; *p* = 0.0589). Thus, the addition of AST and TP boosted the predictive values of models to further verify the profound effect of TP and AST on functional outcomes.

### A novel prognostic model

**FIGURE 3:**
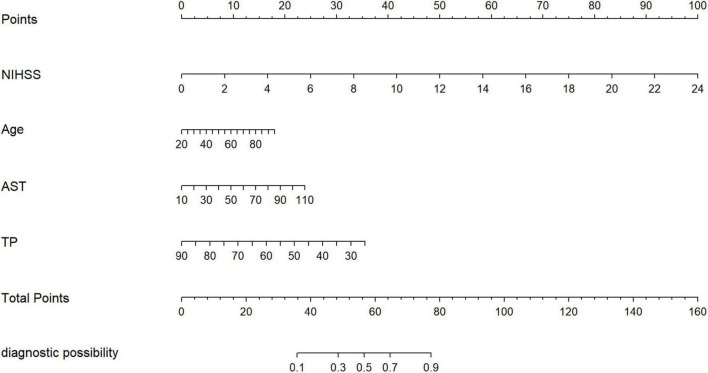
The nomogram for patients with non-CE-type AIS. To use the nomogram, an individual patient’s value is located on each variable axis, and a line is drawn upward to determine the number of points received for each variable value. The sum of these numbers is located on the total points axis, and a line is drawn downward to the survival axes to determine the likelihood of the poor outcomes.

**FIGURE 4:**
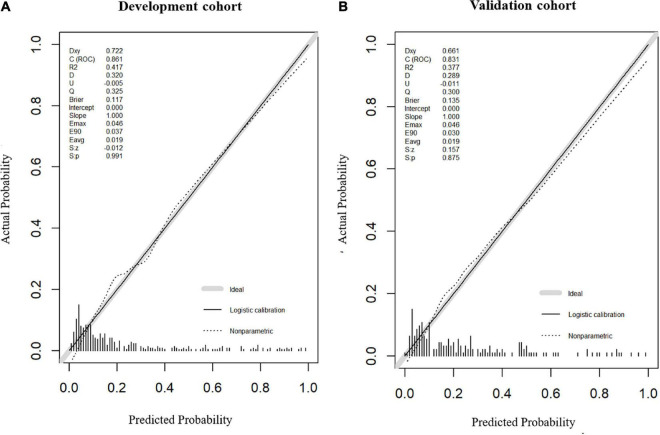
Calibration plot of nomogram in the development cohort and the validation cohort. Calibration curves for the 1-year functional outcomes, which are representative of predictive accuracy, for the development **(A)** and the validation cohorts **(B)**.

Model 2, more convenient and accessible than model 1, was selected as the prediction model. We included liver function predictors (AST and TP) to establish a novel nomogram to predict an unfavorable functional outcome (Figure 3). Furthermore, the prognostic calibration plot for the predictive model in both the development and validation cohorts presented that the predicted 1-year prognosis commendably matched the actual 1-year prognosis. Meanwhile, the C-index values for the nomogram in both the cohorts were 0.861 and 0.831, respectively (Figure 4), illustrating the excellent forecasting ability of liver function indicators. Meanwhile, both the development cohort and the validation cohort had good goodness of fit, with Hosmer–Lemeshow test scores of 0.392 and 0.941, respectively.

### Subgroup analysis of liver function indicators and functional outcome

**FIGURE 5:**
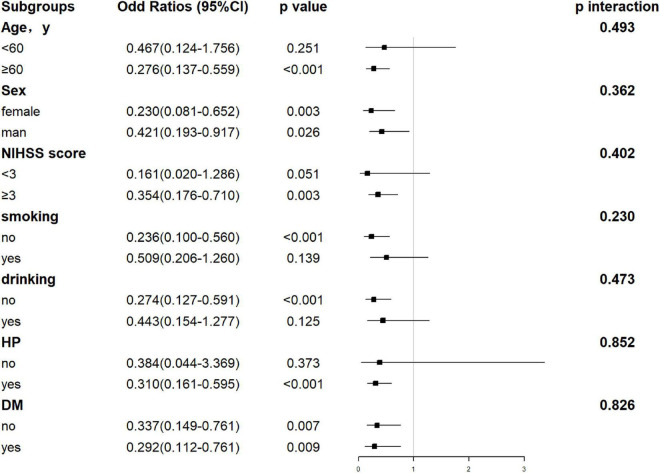
Forest plot of subgroup analysis for the association between TP levels and functional outcome at 1 year. The TP becomes a dichotomous variable with the optimal cutoff value determined according to Youden’s index of the ROC curve. TP is divided into < 68.75 and ≥ 68.75 g/l. NIHSS, National Institutes of Health Stroke Scale; HP, hypertension; DM, diabetes mellitus; TP, total protein.

**FIGURE 6:**
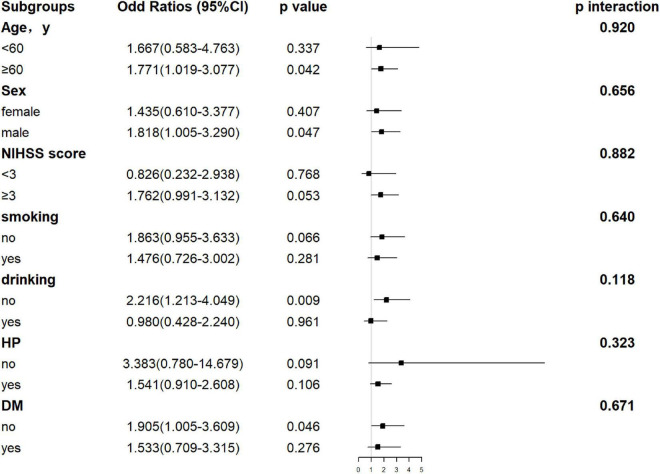
Forest plot of subgroup analysis for the association between AST levels and functional outcome at 1 year. The AST becomes a dichotomous variable with the optimal cutoff value determined according to Youden’s index of the ROC curve. It is divided into < 22.50 and ≥ 22.50 U/l. NIHSS, National Institutes of Health Stroke Scale; HP, hypertension; DM, diabetes mellitus; AST, aspartate aminotransferase.

**FIGURE 7:**
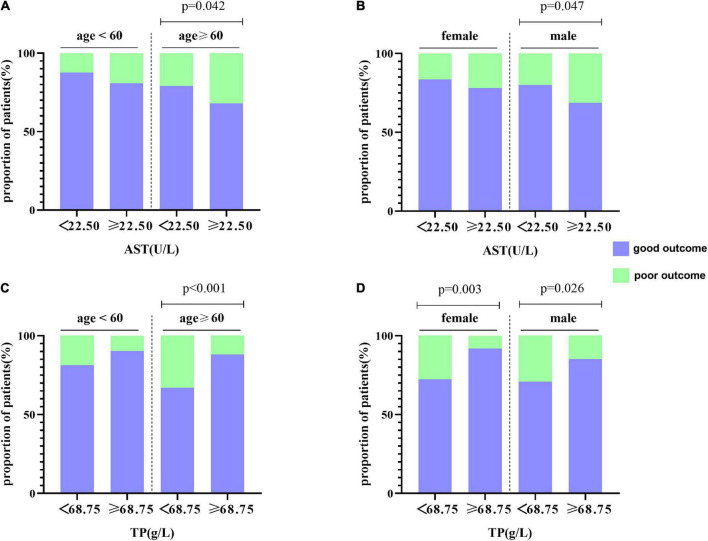
Subgroup analysis for the association between AST, TP, and functional outcome at 1 year. **(A,B)** The distribution of AST is stratified by age and gender, respectively. **(C,D)** The distribution of TP is stratified by age and gender, respectively.

Hepatic function parameters comprising AST as well as TP were converted into dichotomous variables, with the optimal cutoff values determined according to Youden’s index of the ROC curves (Supplementary Table 5). As predictors of functional outcome, optimal cutoff values of AST and TP were 22.50 U/l and 68.75 g/l, respectively. Stratified by age (<60 or ≥60 years), gender (male or female), the NIHSS on admission (<3 or ≥3), cigarette smoking and alcohol consumption, history of hypertension, and diabetes mellitus, subgroup analysis was presented to verify whether AST and TP were still correlated with individuals’ prognosis (mRS). In most groups, a comparison of TP levels conferred a discrepancy that patients with higher TP levels were related to the descending incidence of unfavorable outcomes (Figure 5). Moreover, increased AST levels tended to be associated with an increased risk of unfavorable outcomes in comparison with decreased AST levels. Nevertheless, the results were not suitable for groups with the NIHSS < 3 and alcohol consumption (Figure 6). Furthermore, we revealed that the discrepancy in the levels of TP and AST on different functional outcomes could be discovered in male patients of age ≥ 60 years (Figure 7). Consequently, we investigated that subgroup analyses further confirmed our findings, especially in males aged 60 years or older, and no significant interaction was detected (all *p* interaction > 0.05).

## Discussion

Originated from the forward-looking and continuous stroke registry of the First Affiliated Hospital of Wenzhou Medical University, the development cohort corroborated that increased AST levels and decreased TP levels (both *p* < 0.05) were demonstrated in patients with adverse outcomes, suggesting that liver function factors may be compelling biomarkers for prognosis. On top of that, a nomogram, based on age, the NIHSS, and liver function indicators (AST and TP), was constructed to extrapolate the underlying influence of AST and TP in the prediction of functional outcome. This correlation was consistent with subgroups analyses, especially in male patients of age ≥ 60 years.

Profound explorations as to the mechanisms of mutual interaction between hepatic function and stroke have been still pending. Basic research of hepatic inflammation showed that AIS could activate the pathway of hepatic inflammation *via* catecholamine and cause hepatic insulin resistance, followed by hyperglycemia, generating lesions in hepatic function eventually (24). Additionally, the stimulation of tumor necrosis factor (TNF)-α to the brain or spinal cord injury induced elevated messenger RNA (mRNA) and protein levels of CCL-2 and CXCL-1 in the liver, elicited leukocytosis and neutrophil infiltration to the cerebrum, which thereby enhanced brain inflammation and the central nervous system response to injury (25). Considering that liver function and stroke interact mutually, it is meaningful to figure out whether liver function parameters can predict prognosis in patients with non-CE.

Total protein, deemed to be a biological biomarker of nutritional status, consists of albumin and globulin. In line with our study, a significant association was revealed between nutritional status and the prognosis of patients diagnosed with AIS (26). As discovered by R Palm et al., the concentration of zinc, linked to TP and ALB in cerebrospinal fluid (CSF), had an association with short-term prognosis in patients with acute infarction (27). Consistent with these, in our study, the multivariate logistic regression analysis endorsed that TP could be a promising and protective biomarker for patients with non-CE, and the incremental incidence of poor prognosis decreased by 0.056 for each 1 g/l increase in TP. This correlation persisted in subgroup analysis, which further confirmed the association of functional outcome with TP levels. ALB, a multifunctional non-glycosylated plasma protein and a main component of TP, is synthesized mainly in the liver and contributes to maintaining plasma colloid osmotic pressure and metabolism transport. There is some evidence that decreased serum ALB levels possibly were involved in poor functional outcomes within 24 h of stroke (28). The protective mechanisms of ALB may be: (1) There exists an association between low albumin and chronic systemic inflammation. Sustaining endothelial inflammation can accelerate cellular processes, activate coagulation cascades, obstruct microcirculation, and minimize cerebral perfusion, which aggravates the severity of the stroke and (2) ALB can neutralize the molecules of phosphatidylcholine, derived from circulating phospholipase A2, and reduce cerebral edema in the rat model of stroke (29, 30).

Aspartate aminotransferase, also known as glutamic oxaloacetate transaminase (GOT), is mainly distributed in the myocardium, followed by the liver, skeletal muscle, and kidney, which engages in regulating glutamate metabolism. Our results agreed with Katica-Mulalic A et al. who pointed out that AST was positively correlated with stroke severity (rho = 0.492; *p* = 0.003) and performed that the mean AST of patients treated with the hypothermia group reduced after 24 h (32.50–31.00 IU/ml) (31). In a retrospective study, the elevation of serum AST/ALT at admission was significantly related to a 3-month unfavorable functional outcomes in patients with AIS (32). It is intriguing to note that in the study group, AST levels in serum and CSF were apparently increased, and the elevation of AST in the latter was greater in the hemorrhagic group in comparison with the ischemic group (33). Similarly, our study also concluded that patients with the poor functional outcomes (mRS > 2) were accompanied by ascending levels of AST than those with a good prognosis (mRS ≤ 2) (OR: 1.026). We further verified the relationship between AST levels and stroke prognosis through the subgroup analysis, and the correlation still existed, especially in males 60 years or older. Contrary to our results, a number of prospective studies discovered that AST, regarded as a prognostic and protective biomarker of stroke, mediated the conversion of glutamate to α-ketoglutaric acid and L-aspartic acid or L-alanine, which decreased glutamate levels and finally mitigated the damage of glutamate to brain tissue (34, 35). This may be due to the type of subjects we included (patients with cardioembolism were excluded). What is more, liver function indicators (AST and TP) in the model were included to scrutinize their common influence rather than just a single AST, which partly explained conflicting results within the above studies.

The correlation of stroke prognosis with liver function parameters has remained elusive. Hence, a cohort study with a sample of 576 was supposed to further address the association of functional outcomes with liver function parameters. Model 2 was taken as the optimal model by a comparison of three models and the nomogram was carried out to visualize the results of the logistic regression model. Meanwhile, the predictive ability of the nomogram was well confirmed by the calibration curves. Liver function indicators (AST and TP), whose detection methods are sensitive, standardized, simple, and inexpensive, are considered a set of definite prognostic biomarkers for non-CE, and therefore, hold essential clinical implications.

Still, our study presents some limitations. First of all, we utilized the internal validation and ignored the external validation. George et al. revealed that most of the studies adopt internal validation, and articles, accounting for 75%, were short of external validation (36). Future studies should be involved in the external validation method of multicenter cooperation. Second, other liver function indicators, possibly potential predictors of functional outcome, have not been discovered in our research, incorporating ALT, ALB, and GGT (16, 17). Third, liver function parameters plausibly possessed the change dynamically during admission, which disturbed the prognosis of stroke. Further studies should be focused on dynamic detection to preferably clarify the relationship between liver function and prognosis. Fourth, since the heterogeneity of study samples varies from countries and quantities, it is not straightforward to generalize our results to other countries and populations. Last but not least, a multitude of studies have shown that it is common for researchers to divide the variables, independent or dependent, into four quarters for further explorations (37–39). Nevertheless, we investigated the study on the follow-up data after 1 year and 3 months and were unable to systematically collect data at 3rd, 6th, 9th, and 12th months. Further investigation should be highlighted on the correlation between liver function parameters and functional outcome through following up at first, second, third, and fourth quarters of year.

## Conclusion

In total, liver function indicators might serve as promising and independent biomarkers for patients with non-CE, especially in males aged ≥ 60 years. The increment in AST is positively correlated with the unfavorable functional outcomes at 1-year follow-up. As a neuroprotective prognostic biomarker, lower levels of TP may be associated with an increased risk for poor prognosis in patients with non-CE. What is more, the nomogram based on hepatic function parameters, in combination with the NIHSS scores and age, is a valid and novel instrument with profound and lasting importance for predicting the prognosis of the disease and helps clinicians to make appropriate treatment strategies.

## Data availability statement

The raw data supporting the conclusions of this article will be made available by the authors, without undue reservation.

## Ethics statement

The studies involving human participants were reviewed and approved by the Ethical Decision Committee of the Research Administration at First Affiliated Hospital of Wenzhou Medical University approved the study. The patients/participants provided their written informed consent to participate in this study.

## Author contributions

JX and YZ were responsible for data statistics and writing articles. CP, LG, HY, WL, and WZ collected the data. BD provided resources and designed the study. All authors contributed to the article and approved the submitted version.

## Supplementary material

Supplementary Figure 1 **(A–C)** Correlation between hepatic function parameters and prognosis of patients with non-CE. **(D)** The Spearman’s correlation coefficient between AST, TP, ALB, and other related indexes in patients with non-CE.

Supplementary Figure 2 The ROC curve of the model after a 3-month follow-up. Model: NIHSS + Age + AST + TP + ALB + NLR + fT3 + FI. ROC, receiver operating characteristic.

Supplementary Figure 3 The DCA for three models to predict the correct diagnosis of patients with poor outcomes in **(A)** the development and **(B)** the validation cohorts. The net benefit value of model 1 and model 2 are higher than model 3.DCA, decision curve analysis.

Supplementary Figure 4 Net reclassification index between model 2 and model 3 in **(A)** the development and **(B)** the validation cohorts. NRI, net reclassification index.
