# Supplementary material for: Liver function parameters aspartate aminotransferase and total protein predict functional outcome in stroke patients with non-cardioembolism
Source: Front Nutr. 2022 Aug 18;9:918553. doi: 10.3389/fnut.2022.918553 (PMC9434273; doi:10.3389/fnut.2022.918553)

Supplementary Information

Liver function parameters aspartate aminotransferase and total protein predict functional outcome in stroke patients with non-cardioembolism

Jiali Xie[1,2], Yinmeng Zhu[1,2], Chunyang Pang[1], Lingfei Gao[1], Huan Yu[3], Wenjing Lv[4], Wanli Zhang[1] and Binbin Deng[1]*

1. Department of Neurology, First Affiliated Hospital of Wenzhou Medical University, Wenzhou, China.
2. First Clinical College of Wenzhou Medical University, Wenzhou, China.
3. Department of Pediatrics, Second Affiliated Hospital and Yuying Children's Hospital of Wenzhou Medical University, Wenzhou, China.
4. Department of Geriatrics, The Affiliated Hospital of Qingdao University, Qingdao, China

*Corresponding author: Binbin Deng, postal address: First Affiliated Hospital of Wenzhou Medical University, Wenzhou, PR. China, email address: dbinbin@aliyun.com

Supplementary Table 1. The general characteristics of the total and development cohorts.

Supplementary Table 2. Multivariate logistic regression according to the functional outcome.

Supplementary Table 3. Logistic regression according to the functional outcome after 3-month follow-up.

Supplementary Table 4.NRI and IDI between Model2 and Model3 groups in development and validation cohort.

Supplementary Table 5. ROC curves for unfavorable functional outcome.

Supplementary Figure 1. (**A–C**) Correlation between hepatic parameters and prognosis of non-CE patients. **(D)** Spearman correlation coefficient between AST, TP, ALB and other related indexes in non-CE patients.

Supplementary Figure 2. The ROC curve of the model after a 3-month follow-up. Model: NIHSS + Age + AST + TP + ALB + NLR + fT3 + FI. ROC, receiver operating characteristic.

Supplementary Figure 3. The DCA for three models to predict the correct diagnosis of patients with poor outcomes in (A) the development and (B) the validation cohorts. The net benefit value of model 1 and model 2 are higher than model 3. DCA, decision curve analysis.

Supplementary Figure 4. Net reclassification index between model 2 and model 3 in (A) the development and (B) the validation cohorts. NRI, net reclassification index.

Supplementary **Table 1. The general characteristics of the total and development cohorts**

	Total cohort			Development cohort		
**Group**	**mRS≤2**	**mRS>2**	**p**	**mRS≤2**	**mRS>2**	**p**
**N**	433	134		292	86	
**Age**	63.64±11.782	70.01±10.090	<0.001***	64.54±11.914	69.33±10.481	<0.001***
**SBP**	154.05±23.238	158.17±20.210	0.065	153.51±23.087	156.12±20.549	0.346
**DBP**	81.67±13.782	82.06±13.006	0.769	80.32±12.685	82.00±13.040	0.283
**NIHSS**	2.77±2.241	6.50±4.044	<0.001***	2.57±2.237	6.65±3.910	<0.001***
**Neutrophil**	4.08±1.753	4.83±2.065	<0.001***	4.11±1.848	4.79±1.961	0.004**
**Lymphocyte**	1.88±0.623	1.62±0.629	<0.001***	1.89±0.646	1.60±0.662	0.001***
**NLR**	2.52±2.333	3.49±2.406	<0.001***	2.61±2.731	3.59±2.527	0.003**
**PLT**	222.31±61.247	224.95±67.144	0.671	223.53±61.012	228.41±66.395	0.525
**RBC**	4.52±0.594	4.44±0.503	0.148	4.48±0.603	4.46±0.500	0.774
**HB**	137.48±17.337	133.87±16.556	0.030*	136.50±17.821	134.80±16.790	0.433
**TP**	67.28±5.788	65.78±5.639	0.009**	67.41±5.776	65.25±6.237	0.003**
**ALB**	38.48±3.796	36.75±3.852	<0.001***	38.51±3.927	36.66±3.903	<0.001***
**ALT**	22.74±14.200	21.76±14.208	0.488	22.40±14.921	21.62±15.293	0.673
**AST**	23.62±9.358	27.05±13.998	0.009**	23.50±9.327	27.41±16.222	0.036*
**BUN**	5.05±1.914	5.77±3.996	0.046*	5.15±2.032	5.77±4.498	0.073
**BUN/Cr**	18.08±5.697	18.78±6.331	0.227	18.41±5.802	19.00±6.131	0.420
**ALP**	83.37±26.065	88.41±36.553	0.187	83.45±24.767	91.20±42.406	0.222
**GGT**	42.12±38.778	45.19±54.402	0.591	39.32±35.706	45.90±63.207	0.365
**TB**	11.37±4.914	12.17±6.029	0.122	11.37±5.162	11.78±5.615	0.534
**IB**	7.17±3.366	7.63±4.270	0.207	7.19±3.522	7.50±4.085	0.503
**DB**	4.16±1.897	4.61±2.044	0.019*	4.16±2.039	4.44±1.832	0.255
**Cr**	70.74±20.111	75.32±33.170	0.053	70.65±21.200	75.00±34.884	0.157
**TC**	4.69±1.196	4.49±1.136	0.089	4.72±1.231	4.54±1.196	0.242
**LDL**	2.68±0.881	2.62±0.806	0.475	2.65±0.911	2.65±0.820	0.999
**HDL**	1.09±0.294	1.10±0.321	0.694	1.10±0.272	1.06±0.246	0.228
**TSH**	1.95±2.344	2.06±2.281	0.643	1.88±1.169	2.16±2.494	0.324
**Hcy**	10.80±8.329	11.49±9.218	0.482	10.78±9.089	11.43±9.268	0.626
**TH**	103.57±18.564	106.52±19.348	0.120	103.01±18.663	103.61±17.664	0.796
**fT3**	4.58±0.722	4.27±0.714	<0.001***	4.52±0.630	4.23±0.752	<0.001***

**fT4**	11.20±1.964	11.60±2.300	0.057	11.22±2.074	11.23±2.296	0.986
**FI**	3.54±1.015	3.82±1.246	0.023*	3.54±1.048	3.85±1.209	0.042*
**CK**	98.26±113.234	132.57±203.314	0.090	96.13±62.367	145.63±244.777	0.093
**Sex**			0.572			0.123
female	157（36.3%）	45（33.6%）		115（39.4%）	26（30.2%）	
male	276（63.7%）	89（66.4%）		177（60.6%）	60（69.8%）	
**HP**			0.012*			0.261
no	75（17.4%）	11（8.4%）		50（17.2%）	10（12.0%）	
yes	357（82.6%）	120（91.6%）		241（82.8%）	73（88.0%）	
**AF**			0.026*			0.126
no	413（95.4%）	120（90.2%）		280（95.9%）	78（91.8%）	
yes	20（4.6%）	13（9.8%）		12（4.1%）	7（8.2%）	
**DM**			0.676			0.712
no	266（61.7%）	80（59.7%）		179（61.5%）	51（59.3%）	
yes	165（38.3%）	54（40.3%）		112（38.5%）	35（40.7%）	
**smoking**			0.501			0.165
no	241（55.7%）	79（59.0%）		174（59.6%）	44（51.2%）	
yes	192（44.3%）	55（41.0%）		118（40.4%）	42（48.8%）	
**drinking**			0.479			0.528
no	279（64.6%）	91（67.9%）		200（68.7%）	56（65.1%）	
yes	153（35.4%）	43（32.1%）		91（31.3%）	30（34.9%）	

Abbreviations: mRS Modified Rankin Scale, SBP systolic blood pressure, DBP diastolic blood pressure, NIHSS National Institutes of Health Stroke Scale on admission, NLR neutrophil-to-lymphocyte ratio, PLT platelet, RBC red blood cell, HB hemoglobin, TP total protein, ALB albumin, ALT alanine aminotransferase, AST aspartate aminotransferase, BUN urea nitrogen, BUN/Cr urea creatinine ratio, ALP alkaline phosphatase, GGT gamma-glutamyl transpeptidase, TB total bilirubin, IB indirect bilirubin, DB direct bilirubin, Cr creatinine, TC total cholesterol, LDL low density lipoprotein, HDL high density lipoprotein, TSH thyroid stimulating hormone, Hcy homocysteine, TH thyroid hormones, fT3 free triiodothyronine, fT4 free tetraiodothyronine, FI fibrinogen, CK creatine kinase, HP hypertension, AF atrial fibrillation, DM diabetes mellitus,*p < 0.05,**p < 0.01,***p < 0.001

Supplementary Table 2. Multivariate logistic regression according to the functional outcome

	Model1				Model2				Model3		
	**OR**	**95%CI**	**P**		**OR**	**95%CI**	**P**		**OR**	**95%CI**	**P**
**age**	1.034	1.022-1.067	0.039*	**Age**	1.026	0.999-1.053	0.060	**Age**	1.030	1.003-1.057	0.027*
**NIHSS**	1.613	1.423-1.828	<0.001***	**NIHSS**	1.556	1.393-1.738	<0.001***	**NIHSS**	1.573	1.409-1.755	<0.001***
**TP**	0.918	0.854-0.986	0.019*	**TP**	0.944	0.899-0.991	0.020*				
**AST**	1.029	1.003-1.056	0.027*	**AST**	1.026	1.002-1.050	0.031*				
**NLR**	0.979	0.873-1.097	0.712								
**ALB**	1.099	0.955-1.264	0.188								
**fT3**	0.830	0.485-1.420	0.496								
**FI**	1.068	0.778-1.467	0.684								

Note: Logistic regression analysis found that patients' age, TP, AST and NIHSS are independent risk factors for functional outcome. On this basis, we have established three models, Model 1, NIHSS + age + AST + TP + ALB + NLR + fT3 + FI; Model 2, NIHSS + age + Hepatic parameters (AST and TP); Model 3, NIHSS + age; Abbreviations: NIHSS National Institutes of Health Stroke Scale on admission, NLR neutrophil-to-lymphocyte ratio, TP total protein, ALB albumin, AST aspartate aminotransferase, fT3 free triiodothyronine, FI fibrinogen, *p < 0.05, **p < 0.01, ***p < 0.001

Supplementary Table 3. Logistic regression according to the functional outcome after 3-month follow-up

	Adjusted Model		
	OR	95%CI	P
**Age**	1.067	1.019-1.118	0.006**
**NIHSS**	1.734	1.421-2.115	<0.001***
**TP**	0.898	0.809-0.998	0.045*
**AST**	1.042	1.002-1.085	0.040*
**NLR**	0.788	0.567-1.095	0.156
**ALB**	1.199	0.990-1.452	0.063
**fT3**	0.529	0.243-1.151	0.108
**FI**	0.944	0.602-1.479	0.800

Note: Logistic regression analysis found that patients' age, TP, AST and NIHSS are independent risk factors for functional outcome at 3-month follow up. Adjusted model, NIHSS + age + AST + TP + ALB + NLR + fT3 + FI; Abbreviations: NIHSS National Institutes of Health Stroke Scale on admission, NLR neutrophil-to-lymphocyte ratio, TP total protein, ALB albumin, AST aspartate aminotransferase, fT3 free triiodothyronine, FI fibrinogen, *p < 0.05, **p < 0.01, ***p < 0.001

Supplementary Table 4.NRI and IDI between Model2 and Model3 groups in development and validation cohort

		development cohort	validation cohort
Model2	**IDI**	0.0228 [-0.0011 - 0.0467]	0.0206 [-0.0008 - 0.0421]
	**P value**	0.0611	0.0589
Model3	**NRI**	0.1141 [0.0260 - 0.2021]	0.1423[0.0122- 0.2724]
	**P value**	0.0111	0.0321

Note: Both NRI and IDI in development and validation cohort show that Model2 has a higher predictive power than Model3. Abbreviations: NRI: Net Reclassification Index; IDI: Integrated Discrimination Improvement;

Supplementary Table 5. ROC curves for unfavorable functional outcome

	AST	TP
AUC	0.553	0.592
95% CI	0.478-0.627	0.528-0.656
optimal cutoff value	22.5	68.75
specificity	0.426	0.622
sensitivity	0.558	0.837
Youden index	0.133	0.216

Abbreviations: ROC, receiver operating characteristic; AUC, area under curve; CI, confidence interval; AST, aspartate amino transferase; TP, total protein.

Supplementary Figure 1. (**A–C**) Correlation between hepatic parameters and prognosis of non-CE patients. **(D)** Spearman correlation coefficient between AST, TP, ALB and other related indexes in non-CE patients.

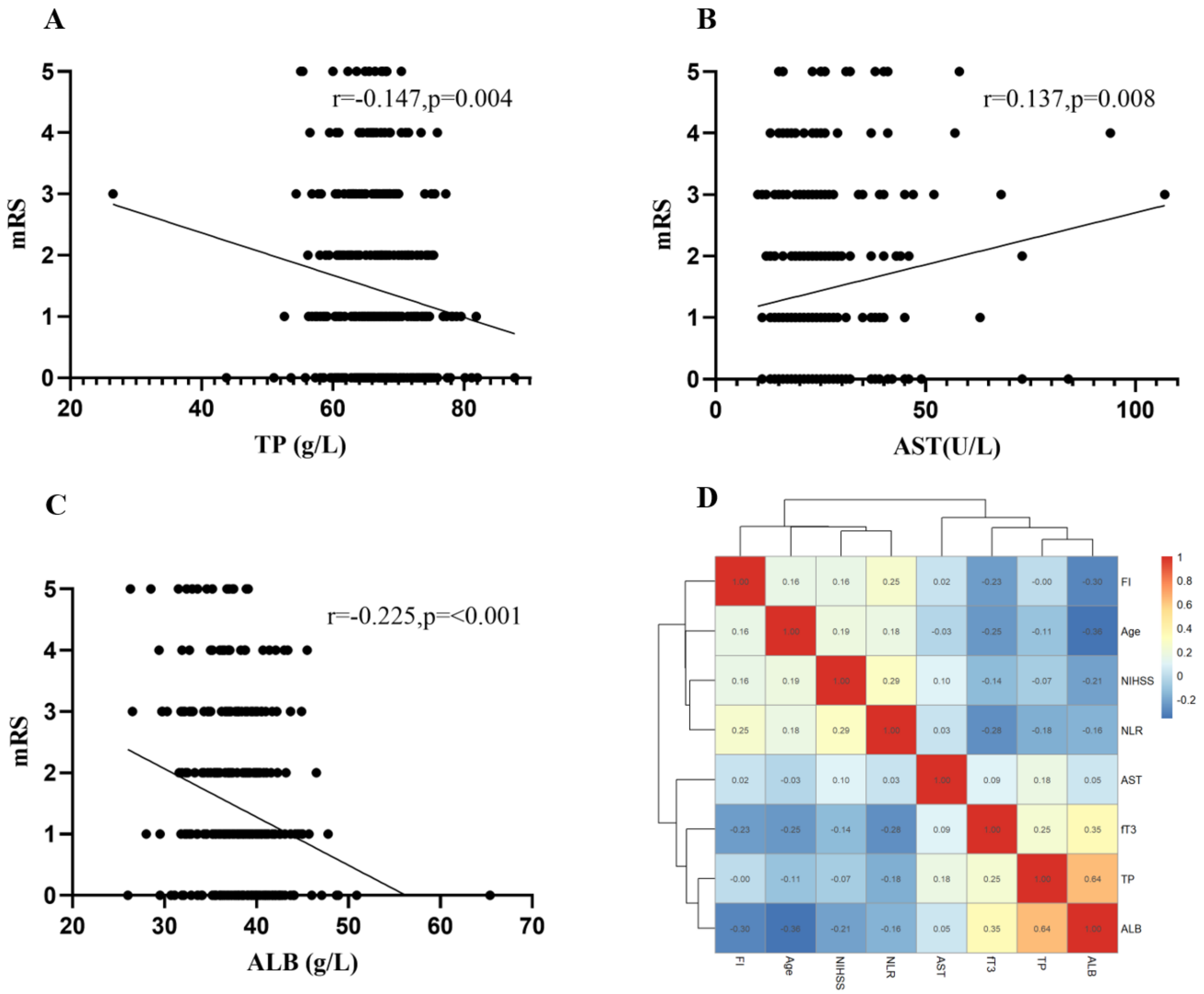

Supplementary Figure 2. The ROC curve of the model after a 3-month follow-up. Model: NIHSS + Age + AST + TP + ALB + NLR + fT3 + FI. ROC, receiver operating characteristic.

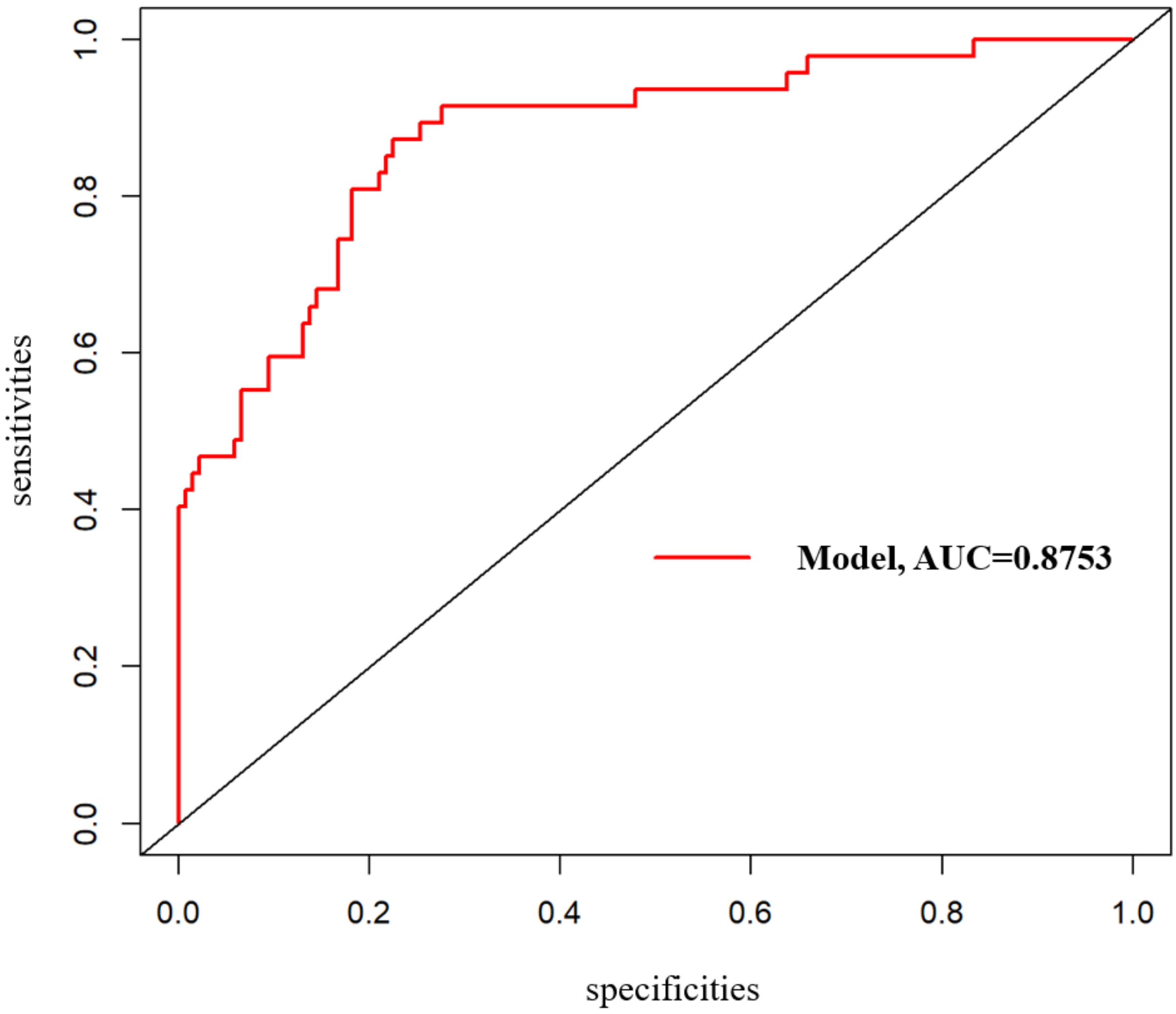

Supplementary Figure 3. The DCA for three models to predict the correct diagnosis of patients with poor outcomes in (A) the development and (B) the validation cohorts. The net benefit value of model 1 and model 2 are higher than model 3. DCA, decision curve analysis.

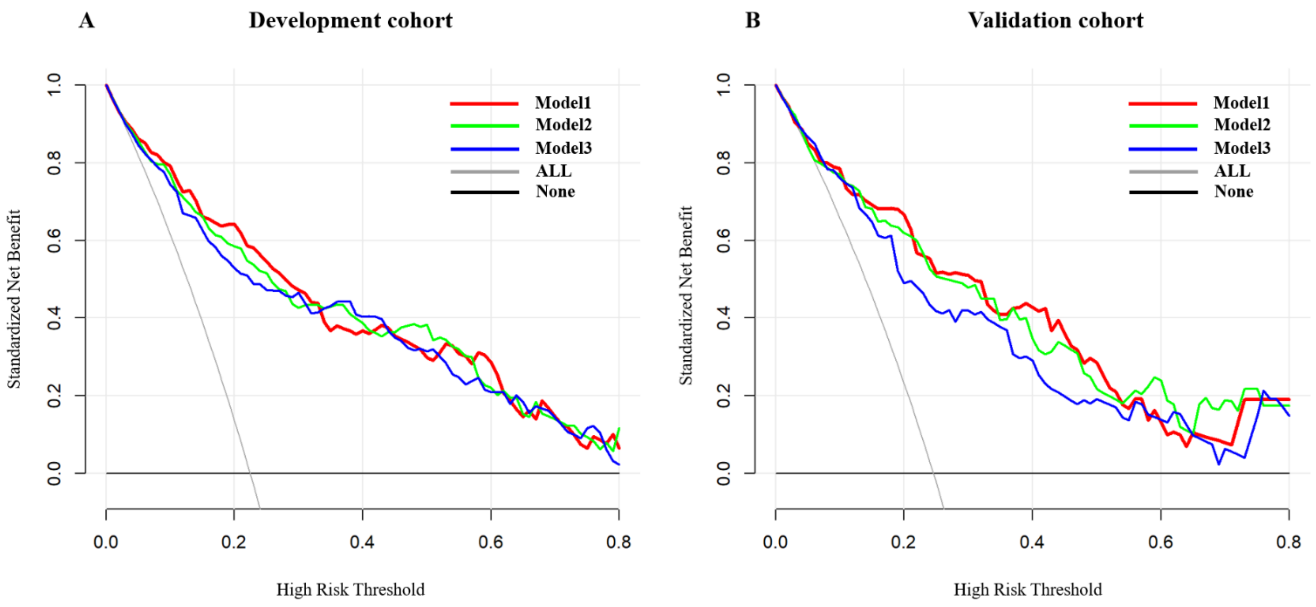

Supplementary Figure 4. Net reclassification index between model 2 and model 3 in (A) the development and (B) the validation cohorts. NRI, net reclassification index.

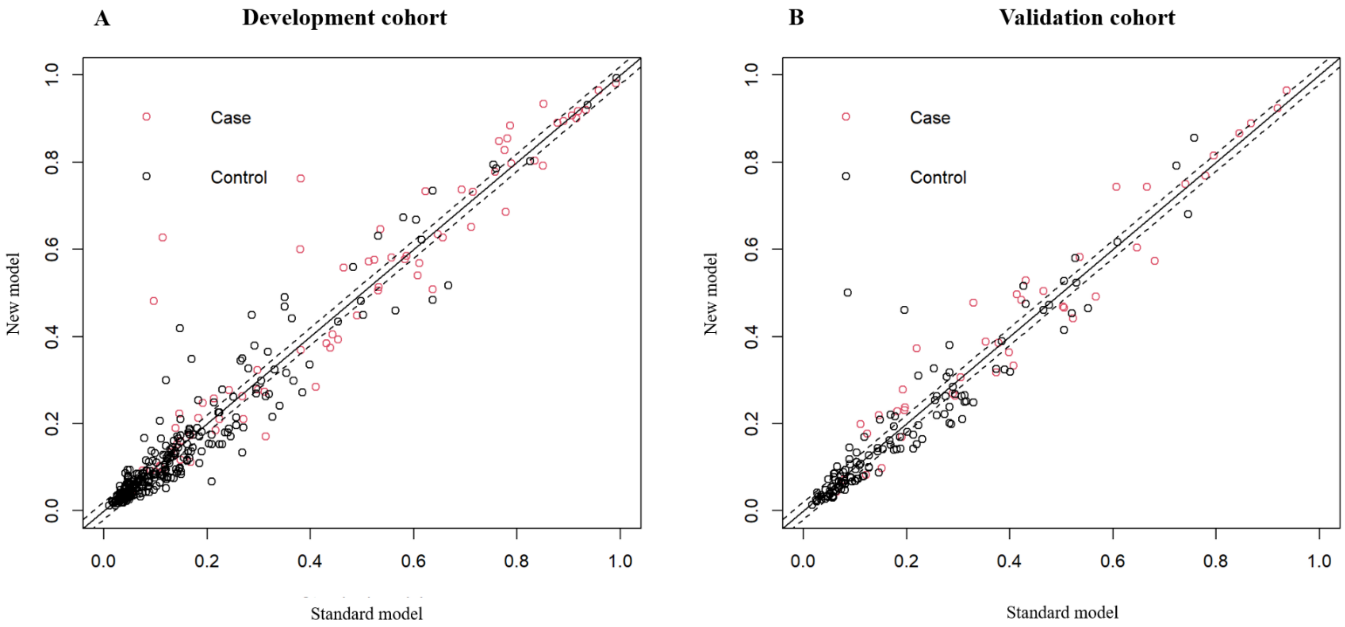

Supplement: Supplementary Figure 1 — (A–C) Correlation between hepatic function parameters and prognosis of patients with non-CE. (D) The Spearman’s correlation coefficient between AST, TP, ALB, and other related indexes in patients with non-CE. [file Data_Sheet_1.pdf]
